# Intracranial Lesion Detection and Artifact Characterization: Comparative Study of Susceptibility and T2^*^-Weighted Imaging in Dogs and Cats

**DOI:** 10.3389/fvets.2021.779515

**Published:** 2021-12-13

**Authors:** Nadja Wolfer, Adriano Wang-Leandro, Katrin M. Beckmann, Henning Richter, Matthias Dennler

**Affiliations:** ^1^Clinic for Diagnostic Imaging, Department of Clinical Diagnostics and Services, Vetsuisse Faculty, University of Zurich, Zurich, Switzerland; ^2^Section of Neurology and Neurosurgery, Small Animal Clinic, Vetsuisse Faculty, University of Zurich, Zurich, Switzerland

**Keywords:** MRI, SWI, calcification, CT, canine, feline, hemorrhage

## Abstract

Susceptibility-weighted imaging (SWI), an MRI sequence for the detection of hemorrhage, allows differentiation of paramagnetic and diamagnetic substances based on tissue magnetic susceptibility differences. The three aims of this retrospective study included a comparison of the number of areas of signal void (ASV) between SWI and T2^*^-weighted imaging (T2^*^WI), differentiation of hemorrhage and calcification, and investigation of image deterioration by artifacts. Two hundred twelve brain MRIs, 160 dogs and 52 cats, were included. The sequences were randomized and evaluated for presence/absence and numbers of ASV and extent of artifacts causing image deterioration by a single, blinded observer. In cases with a CT scan differentiation of paramagnetic (hemorrhagic) and diamagnetic (calcification) lesions was made, SWI was performed to test correct assignment using the Hounsfield Units. Non-parametric tests were performed to compare both sequences regarding detection of ASV and the effect of artifacts on image quality. The presence of ASV was found in 37 SWI sequences and 34 T2^*^WI sequences with a significant increase in ASV only in dogs >5 and ≤ 15 kg in SWI. The remaining weight categories showed no significance. CT examination was available in 11 cases in which 81 ASV were found. With the use of phase images, 77 were classified as paramagnetic and none as diamagnetic. A classification was not possible in four cases. At the level of the frontal sinus, significantly more severe artifacts occurred in cats and dogs (dogs, *p* < 0.001; cats, *p* = 0.001) in SWI. The frontal sinus artifact was significantly less severe in brachycephalic than non-brachycephalic dogs in both sequences (SWI, *p* < 0.001; T2^*^WI, *p* < 0.001). In conclusion, with the advantages of better detection of ASV in SWI compared with T2^*^WI and the opportunity to differentiate between paramagnetic and diamagnetic origin in most cases, SWI is generally recommended for dogs. Frontal sinus conformation appears to be a limiting factor in image interpretation.

## Introduction

Susceptibility-weighted imaging (SWI), a state-of-the-art MRI sequence for hemorrhage detection, has been recently introduced to veterinary medicine (1–4). As a fully velocity-compensated, high-resolution, three-dimensional, gradient-echo sequence, SWI enables detection of paramagnetic blood products (deoxyhemoglobin, methemoglobin, or hemosiderin) and diamagnetic calcifications (5, 6). Paramagnetic substances can increase the local magnetic field strength, while diamagnetic substances decrease the local magnetic field strength. In contrast to conventional T2^*^-weighted imaging (T2^*^WI), SWI may distinguish paramagnetic substances from calcification (diamagnetic) (7–9) with increased sensitivity for detection of small brain parenchymal hemorrhages (10–12). SWI encompasses magnitude and filtered phase images and fuses both different image types for evaluation; however, phase images can also be evaluated independently. Hemorrhage and calcification are recognized as areas of signal void (ASV) in the SWI. Phase images are useful to discriminate between paramagnetic and diamagnetic substances (7–9). The appearance of a lesion, the basis for identification and classification as hemorrhage or calcifications, is influenced by the handedness of the scanner. Depending on whether the MRI system is right- or left-handed, a characteristic that varies among vendors, the lesion appears differently (i.e., opposite). Therefore, for correct interpretation of the images, knowing the handedness of the MRI system is mandatory. In a right-handed system, paramagnetic substances are hypointense and diamagnetic substances are hyperintense and vice versa in a left-handed magnetic field (6, 7, 13).

Apart from hemorrhage and calcification, metal implants (e.g., microchip) (14) and normal anatomical structures can cause distortions of the magnetic field (13). This causes artifacts in SWI, especially at bone–air interfaces of the sinuses and the petrosal portion of the temporal bone (15, 16), which are reinforced with increasing field strength (17, 18). These susceptibility artifacts have the potential to mimic non-existent blood or blood products in the adjacent brain parenchyma or to mask subtle foci of susceptibility in these areas (17). Compared with humans, dogs and cats have a smaller brain volume (19–21) and larger frontal sinuses, which may make them more prone to the abovementioned artifacts. Absent or minuscule air-filled frontal sinuses are known in some small-breed dogs, including many brachycephalic breeds (22). The current literature describing the use of SWI in veterinary medicine reports field strength of 1.5 Tesla (T) (1, 3). In human medicine, lesion contrast and venous vessel contrast are increased in 3 compared with 1.5 T; however, susceptibility artifacts caused by anatomical structures are also more prominent (17).

In human neuroimaging, SWI is used predominantly for the evaluation of intracranial hemorrhage, vascular malformations, traumatic brain injury, brain tumors, congenital, infections, and neurodegenerative disorders (6, 7, 15). In veterinary medicine, the utility of SWI sequence has been proven in dogs affected by traumatic brain injury and for detecting hemorrhage and identifying intracranial venous abnormalities (1, 3). A recently published study demonstrated that venous structures and hemorrhagic lesions are more conspicuous in SWI than in T2^*^WI in dogs (3), but studies in cats as well as the influence of unwanted artifacts on the image evaluation are currently lacking.

The aim of this study is threefold: first, to compare detection of intracranial ASV in dogs and cats, between SWI and T2^*^WI; second, to assess the capability of SWI phase images to discriminate between intracranial diamagnetic (calcification) and paramagnetic (hemorrhagic) lesions; and third, to compare the performance of SWI with that of T2^*^WI in cats and dogs according to their weight categories and skull conformation in dogs, considering the susceptibility artifacts caused by normal anatomy.

We hypothesize that more intracranial ASV can be detected with SWI in comparison with T2^*^WI. Furthermore, we hypothesize that SWI can reliably distinguish hemorrhage and calcification and that susceptibility artifacts originating from anatomical structures are more prone in animals with low body weight and non-brachycephalic skull conformation.

## Materials and Methods

### Study Population

Brain MRI studies of cats and dogs acquired between June 2016 and September 2017, at the Clinic for Diagnostic Imaging, Vetsuisse Faculty of the University of Zurich, were retrospectively reviewed. Animals were included if both T2^*^WI and SWI sequences were acquired in the same MRI protocol. Follow-up MRI studies of animals that received more than one MRI study in this period were excluded.

The medical records of the included population were searched for species, breed, age, body weight, sex, and additional CT scan.

### Image Acquisition

MRI was performed under general anesthesia with a 3-T magnetic field scanner using (Philips Ingenia, Philips AG, Zurich, Switzerland; right-handed), a 20-channel head/neck head coil, or an eight-channel small extremity coil (Philips Ingenia, Philips AG, Zurich, Switzerland).

Image parameters of the 2D T2^*^WI sequence included repetition times (TRs) 605–917 ms, dependent on the size of the field of view, echo time (TE) 16 ms, flip angles 18°, and slice thickness 2.5–3 mm. For 3D SWI, TR was 31 ms, TE first 7.2 ms, ΔTE = 6.2 ms, four echoes, flip angle 17°, and slice thickness 2 mm. Both sequences were acquired in transverse planes.

The remaining sequences included gradient-echo T1-weighted, turbo spin-echo T2-weighted, and fluid attenuation inversion recovery.

The CT examinations were performed with a 16-slice scanner (Philips Brilliance 16, Philips AG, Zurich, Switzerland).

### Data Analysis

Sequences were anonymized and randomized. A single evaluator (NW), blinded to the signalment, clinical information, and additional MRI findings, reviewed the SWI and T2^*^WI independently on a DICOM viewer software (HOROS, version 3.3.5, https://horosproject.com) in transverse planes. The images were evaluated for the presence of intracranial ASV and image distortion.

In order to evaluate the influence of body weight on image distortion and detection of intracranial ASV, the animals were divided into species as well as weight categories. The dogs were divided into three weight categories: ≤ 5, >5 to ≤ 15, and >15 kg. The cats were divided into two weight categories: ≤ 5 and >5 kg. Dogs were further divided into brachycephalic and non-brachycephalic groups according to their breed (19, 23).

Sequences with ASV present were further divided into six groups. Examinations with one to five ASV formed groups according to the number of lesions, and those with more than five lesions were combined into an additional, “multiple” group. All ASV clearly assigned to the falx cerebri were not rated.

**Figure 1:**
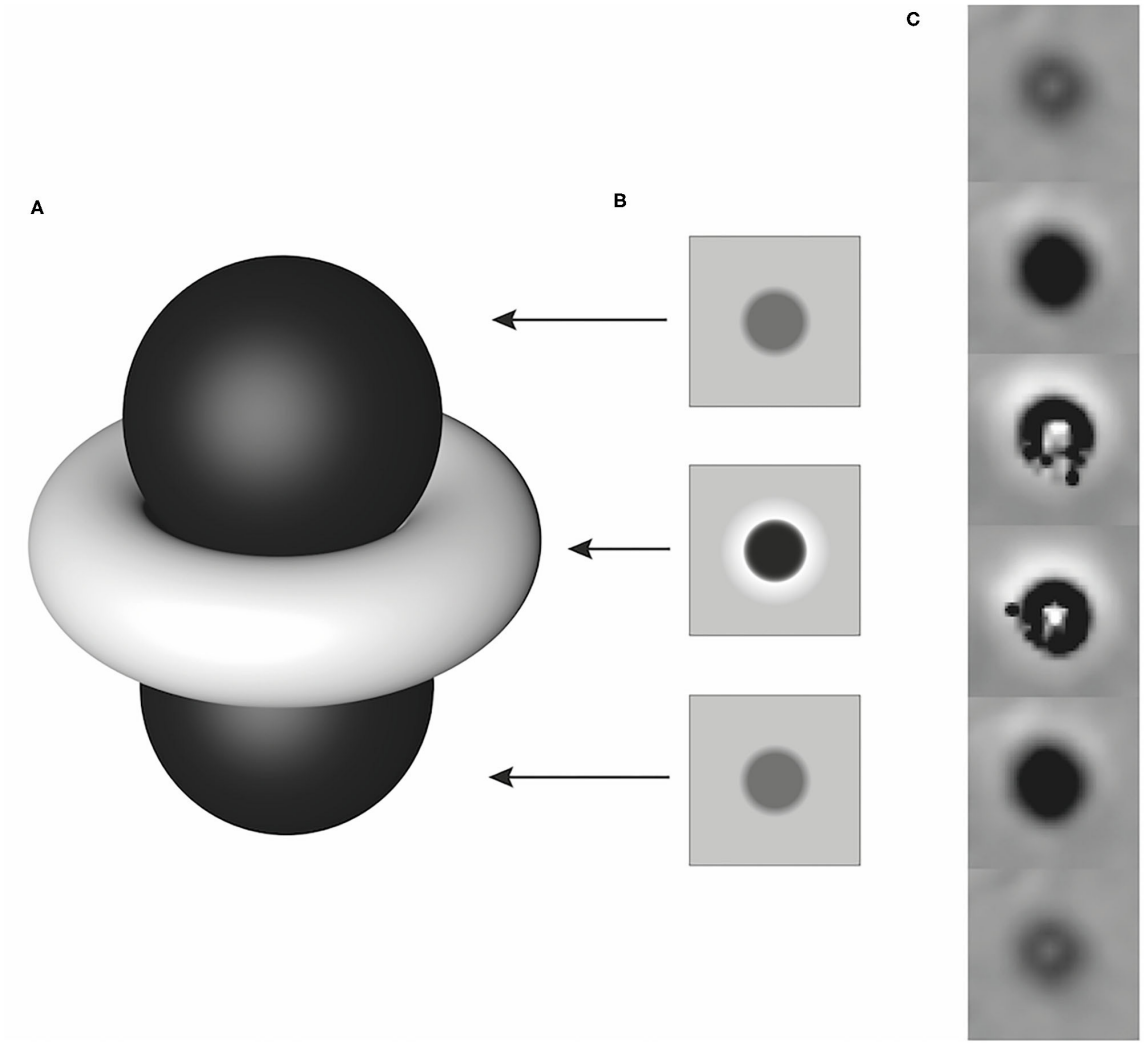
Schematic illustration of a dipole artifact **(A)** with simulated phase images corresponding to the local field distribution in planes above, at the equator, and below the paramagnetic lesion **(B)** and an example of its representation of a paramagnetic lesion in a right-handed system over several consecutive slices **(C)**. Modified according to Deistung et al. (8).

**Figure 2:**
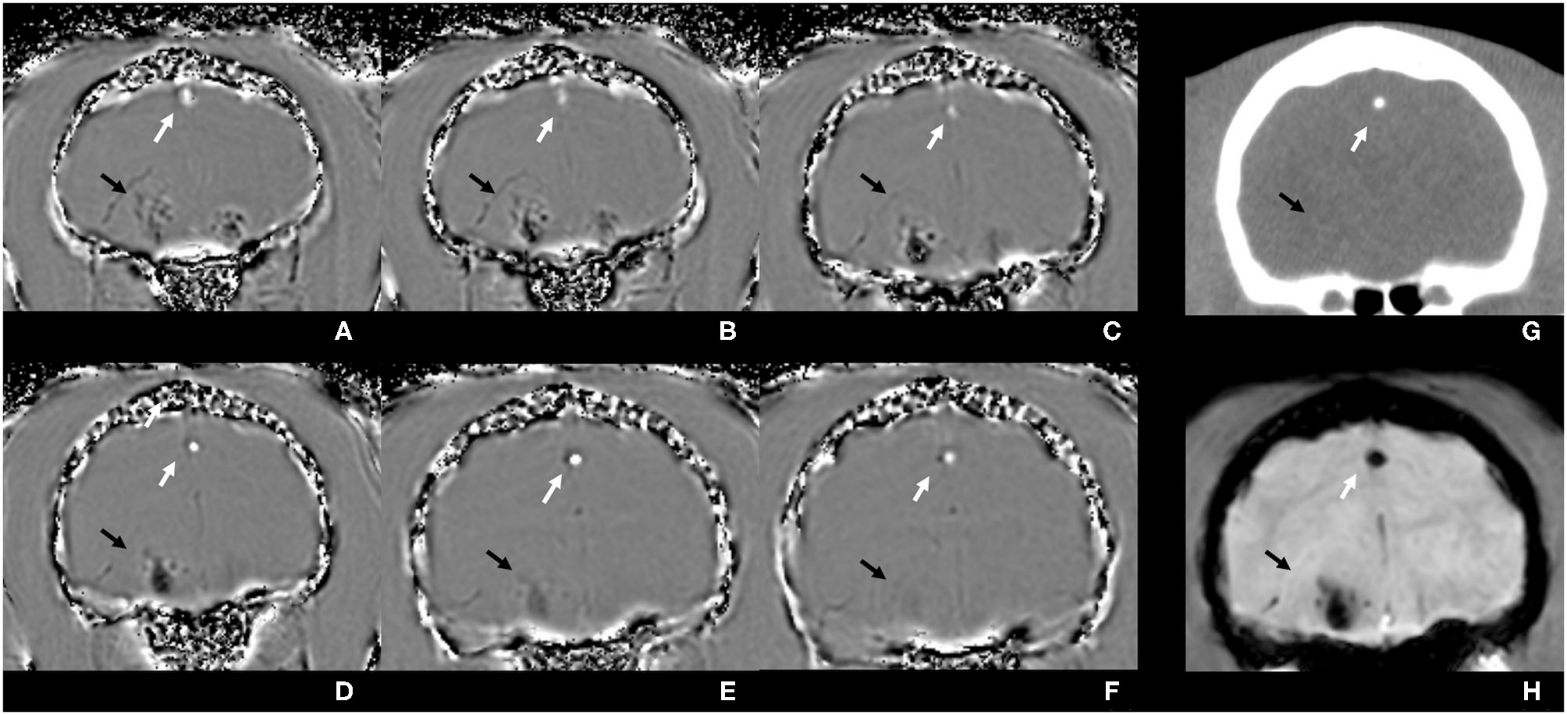
Susceptibility-weighted phase images **(A–F)** of the brain of a cat from the level of the frontal lobes to the level of the tympanic bullae, illustrating signal intensity differences between diamagnetic and paramagnetic tissues. The calcified falx cerebri, diamagnetic, is depicted to be predominantly hyperintense (white arrows), whereas the intracranial hemorrhagic lesion, paramagnetic, is heterogeneous and predominantly hypointense (black arrows). Corresponding CT image in a soft tissue algorithm **(G)**: calcification is highly hyperattenuating (white arrow), and the hemorrhage is iso- to hyperattenuating compared with the brain parenchyma (black arrow). In the susceptibility-weighted magnitude image **(H)**, both lesions have hypointense signal intensity.

All SWI images with the presence of ASV and available corresponding CT studies were assessed for discrimination of calcification and blood products. CT images were used as the gold standard for the absence or presence of calcification. Based on the phase pattern of the dipole effect of a nearly spherical structure, the lesions were identified as paramagnetic and diamagnetic areas and assigned to the hemorrhage and calcification groups accordingly. In a right-handed system, a paramagnetic lesion is characterized by a hyperintense torus and two hypointense lobes (Figure 1) in the phase images (8). As the examinations were performed with a right-handed system, the lesions were assessed as follows: all lesions that presented as hypointense or predominantly hypointense were considered as hemorrhage. Lesions that were hyperintense or predominantly hyperintense were interpreted as calcification (Figure 2). For the larger and more complex lesions, the distinction was made based on the signal behavior over multiple, continuous slices. If a hyperintense torus with two hypointense lobes could be identified, the lesion was counted as hemorrhage. Conversely, a hypointense torus with hyperintense lobes was interpreted as calcification. Lesions that could not be differentiated based on these criteria were considered to be too heterogeneous (7, 9, 13). The CT images were reviewed in a bone and soft tissue algorithm with the following window settings (window width/window level): bone (4,000/1,300) and soft tissue (400/40). An intracranial ASV with a corresponding area of high attenuation [Hounsfield Units (HU) ≥ 100] in CT was interpreted as calcification, whereas ASV matching areas with low attenuation (HU < 100) were counted as bleeding of different stages.

**Figure 3:**
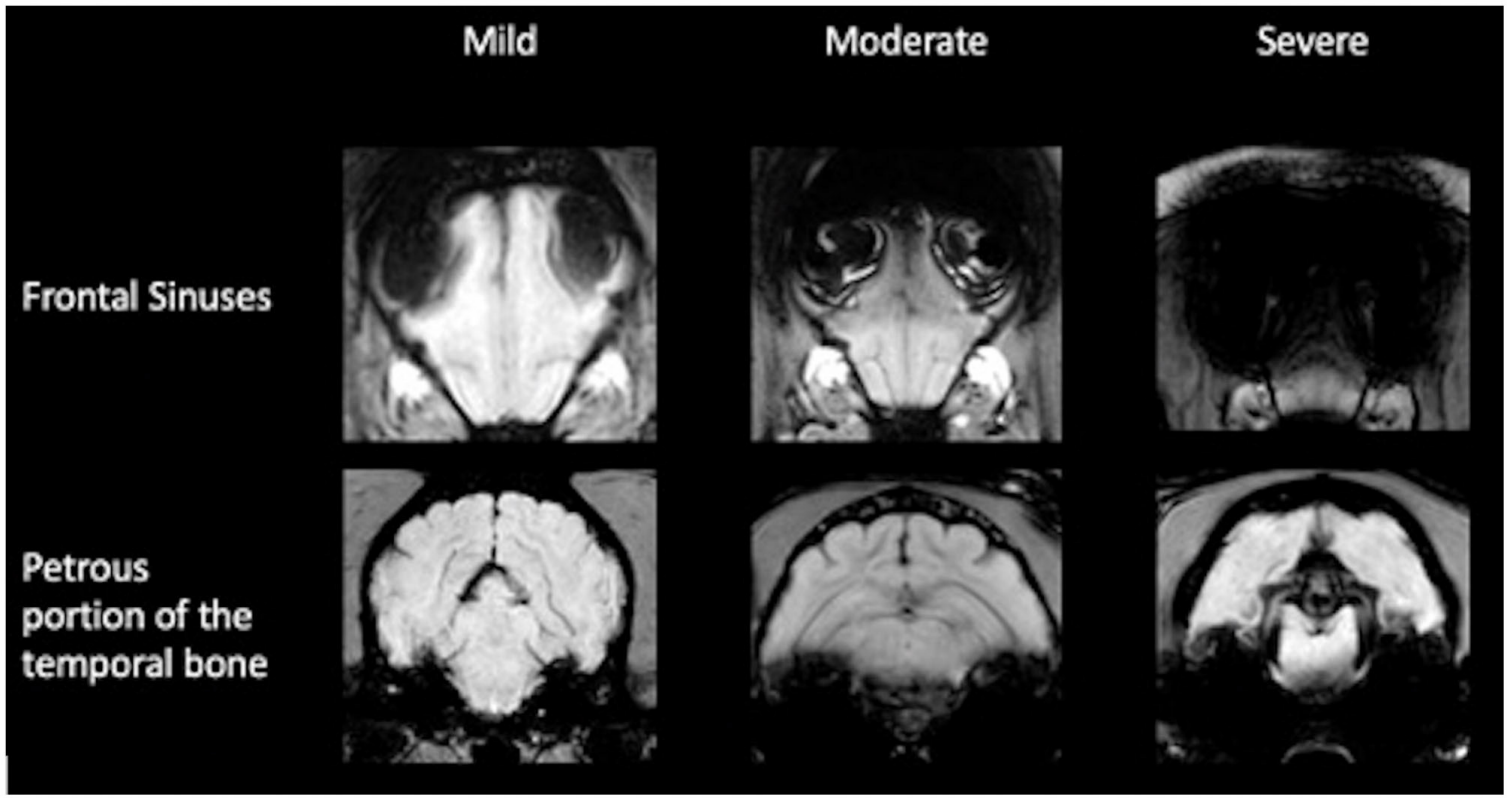
Classification of image distortion at the level of the frontal sinuses and petrous portion of the temporal bone in susceptibility-weighted imaging.

The impairment of the assessment of the sequence in the intracranial region by susceptibility artifact from the frontal sinuses and the petrosal portion of the temporal bone was subjectively evaluated. To assess the artifact, originating from the frontal sinus, the slice halfway between the most rostral portion of the brain parenchyma and the optic chiasm was subjectively graded. If the brain parenchyma at this level was affected up to one-third, it was considered as mild; over one-third to one-half as moderate; and more than one-half as. The artifacts originating from the bullae and petrosal portion of the temporal bone were assessed at the rostral aspect of the bullae. The most dorsal extent of the artifact was related to the intracranial height and classified accordingly: mild up to one-third, moderate up to one-half, and severe at more than one-half (Figure 3). Furthermore, the number of cases in which the microchip caused intracranial image distortion was recorded. Image distortion due to the microchip was not analyzed statistically because the exact localization of the microchip in relation to the vertebrae or the skull could not be evaluated in the examinations of the head.

### Statistical Analysis

Statistical analysis was performed with SPSS (IBM SPSS Statistics, version 25, 64-bit-version, IBM, Chicago, IL, USA). Data were assumed to be not normally distributed due to the limited number of available cases. Descriptive statistics was performed, and quantitative data were displayed with median (range). Dependent groups were compared using the non-parametric Wilcoxon signed-rank test independent groups using the Mann–Whitney U-test (Wilcoxon rank-sum test). Accordingly, SWI and T2^*^WI were defined as dependent groups and were compared for artifacts within the whole population of dogs (*n* = 160) or cats (*n* = 52) or within their subclasses. Subclasses were defined by three weight categories for dogs (≤ 5, >5 to ≤ 15, and >15 kg) and two weight categories for cats (≤ 5 and >5 kg). Another subclass for dogs was determined by skull formation (brachycephalic vs. non-brachycephalic as the independent variable). Overall, a *p*-value of < 0.05 was considered to be significant.

## Results

### Population

A total of 214 MRI studies included both SWI and T2^*^WI. Two dogs underwent follow-up scans; in these two cases, only the first MRI study was included. Therefore, a total of 212 examinations were included, and 52 cats and 160 dogs were further evaluated.

The age in dogs ranged from 3 months to 15 years, with a median age of 7.2 years. The median age in cats was 8.4 years and ranged from 5 months to 16 years. The median weight was 13.2 kg in dogs and 4.3 kg in cats and ranged from 2 to 8.5 kg in cats and from 1.4 to 54 kg in dogs. The gender distribution of the dogs was 50% for both. Of the female dogs, 65% (52/80) and 47.5% (38/80) of the male dogs were neutered. Among the cats, there were 42.3% (22/52) female and 57.7% (30/52) male, of which 90.9% (20/22) of the female cats and 93.3% (28/30) of the male cats were neutered.

Among the population of dogs, 29 belonged to the weight categories ≤ 5, 62 to >5 to ≤ 15 kg, and 69 to >15 kg. In the feline weight category of ≤ 5 kg, there were 32 animals, and in the weight category of >5 kg, 20 animals.

The dog population consisted of 52 brachycephalic and 82 non-brachycephalic dogs. For 26 mix-breed dogs, no assignment to the categories was possible.

### Areas of Signal Void

Intracranial ASV were identified in 45 animals (37 SWI sequences and 34 T2^*^WI sequences). The presence of ASV was detected in 22 dogs and four cats, in both SWI and T2^*^WI. Agreement regarding the number of ASV occurred in 15 dogs and three cats and disagreement in seven dogs and one cat (Table 1; Supplementary Table S1).

**Table 1:** Classification of the number of animals according to the presence and/or absence of areas of signal void in susceptibility-weighted imaging (SWI) and T2*-weighted-imaging (T2*WI).

	**Dogs**	**Cats**	**Total**
	**(*n =* 160)**	**(*n =* 52)**	**(*n =* 212)**
Absence in both	124	43	167
Presence in both	22	4	26
SWI = T2^*^WI	15	3	18
SWI > T2^*^WI	6	1	7
SWI < T2^*^WI	1	0	1
Mismatch	14	5	19
Only SWI, no T2^*^WI	8	3	11
Only T2^*^WI, no SWI	6	2	8

In 27 animals (27/45; 21 dogs/6 cats), the number of ASV differed between sequences. In 14 dogs and five cats, ASV could only be identified in one of the two sequences. The presence of ASV was identified in SWI and not in the corresponding negative T2^*^WI sequence in 11 animals (11/27; 8 dogs/3 cats) and vice versa in eight cases (8/27; 6 dogs/2 cats). A higher number of ASV were identified in the SWI than in the T2^*^WI in 18 cases (18/27; 14 dogs/4 cats) and vice versa in nine cases (9/27; 7 dogs/2 cats). The most common finding was a single ASV in both dogs and cats, in the SWI as well as in the T2^*^WI. The second most common finding in dogs was “multiple” ASV in both sequences. In cats, no more than two ASV were detected in SWI, and only single ASV were detected in T2^*^WI.

Overall, there were no significant differences in the number of ASV detected in dogs and cats between T2^*^WI and SWI (*p* = 0.085 and *p* = 0.317, respectively). When divided into weight categories, a statistically significant higher amount of ASV was detected in SWI relative to T2^*^WI in the population of dogs >5 to ≤ 15 kg (*p* = 0.046). For the other weight categories, no statistical significance was found (≤ 5 kg, *p* = 0.157; >15 kg, *p* = 0.327). No differences were found regarding the detection of lesions between T2^*^WI and SWI in the feline population when categorized by weight (≤ 5 kg, *p* = 1; >5 kg, *p* = 0.180).

### Identification of Intracranial Calcifications Compared With CT

Eleven animals with intracranial ASV identified in SWI had an additional CT examination (2 cats and 9 dogs). CT scans were performed within 0–4 days before or after the MRI studies. A single ASV was present in six animals (5 dogs/1 cat), two ASV were present in one cat, three ASV in one dog, and multiple ASV in three dogs. There were a total of 81 lesions. In phase images, 65 lesions were hypointense or lesions in which the hypointense component was highly predominant. The remaining 16 lesions showed heterogeneous signal intensity in the phase images with 12 of these being classified based on their appearance over consecutive slices showing a hyperintense torus with two hypointense lobes. Therefore, based on the phase images, 77 lesions were classified as hemorrhages. For four lesions, the phase images did not reveal a clear classification due to the geometry and heterogeneity of the lesion.

When comparing the CT images acquired in the same population, no corresponding calcification could be detected. A total of nine lesions (9/81) could be detected in the CT images as hyperattenuating areas relative to the remaining brain parenchyma. Their HU ranged from 26.2 to 65.8 with an SD ranging from ±7 to ±11.5.

### Image Distortion

In the canine population (*n* = 160), image distortion at the frontal sinuses was graded as mild in 78, moderate in 28, and severe in five dogs in T2^*^WI. The frontal sinus did not cause any artifact in the T2^*^WI in 49 cases. At the petrosal portion of the temporal bone, image distortion was graded as mild in all dogs in T2^*^WI. The microchip caused intracranial image distortion in nine dogs in T2^*^WI.

The frontal sinus did not cause any artifact in the SWI in 43 dogs. Image distortion in SWI at the frontal sinuses was graded as mild in 45, moderate in 35, and severe in 37 dogs in SWI. At the petrosal portion of the temporal bone, image distortion in SWI was once classified as moderate, and the remaining image distortions were classified as mild. The microchip caused intracranial image distortion in SWI in 10 dogs.

In the 52 cats, image distortion at the frontal sinuses was graded mild in 17 cats, moderate in 23 cats, and severe in 11 cats in T2^*^WI; and mild in four cats, moderate in 21 cats, and severe in 26 cats in SWI. A single cat showed no image distortions at the level of the frontal sinus in T2^*^WI and SWI. At the petrosal portion of the temporal bone, susceptibility artifacts were graded as mild in 38 and moderate in 14 cats in T2^*^WI, and mild in 28 cats, moderate in 23 cats, and severe in one cat in SWI. Image distortion due to the microchip was identified in two cats in T2^*^WI and in three cats in SWI.

In dogs, SWI revealed significantly increased image distortion in comparison with T2^*^WI at the level of the frontal sinuses in the whole population and in all weight classes (Table 2). In cats, similar findings were present at the level of the frontal sinuses in the whole population and in both weight classes. Additionally, significantly increased image distortion in SWI relative to T2^*^WI was found at the level of the petrosal portion of the temporal bone in the whole population of cats.

**Table 2:** Comparison of image distortion (artifacts ordinally scored as mild, moderate, and severe) between susceptibility-weighted imaging and T2*-weighted imaging for species (dogs and cats) and subclasses defined by weight categories and skull conformation (Wilcoxon test, level of significance, *p* < 0.05).

	**Localization**	
	**Frontal sinuses**	**Petrosal portion of**
		**the temporal bone**
**Dogs**		
Whole population (*n =* 160)	<0.001	0.317
≤ 5 kg (*n =* 29)	0.025	1
>5 to ≤ 15 kg (*n =* 62)	<0.001	1
>15 kg (*n =* 69)	<0.001	0.317
Brachycephalic (*n =* 52)	0.011	1
Non-brachycephalic (*n =* 82)	<0.001	0.317
**Cats**		
Whole population (*n =* 52)	0.001	0.012
≤ 5 kg (*n =* 32)	0.001	0.083
>5 kg (*n =* 20)	0.002	0.059

Dogs show significantly increased image distortion at the level of the frontal sinus in non-brachycephalic as compared with brachycephalic dogs in both sequences (SWI, *p* < 0.001; T2^*^WI, *p* < 0.001). Image distortion at the level of the frontal sinus in the SWI was significantly increased compared with the T2^*^WI in non-brachycephalic and brachycephalic dogs (brachycephalic, *p* = 0.011; non-brachycephalic, *p* < 0.001). There is no significant difference in image distortion between brachycephalic and non-brachycephalic dogs caused by the petrosal bone.

## Discussion

In the present study, a slightly increased number of ASV were detected with the SWI sequence in comparison with the T2^*^WI sequence, particularly in cases with multiple ASV. Increased detection of ASV in SWI was only significant for the group of dogs >5 to ≤ 15 kg. This weight category was most affected by multiple ASV, compatible with microbleeds. In humans, microbleeds are also detected more reliably with SWI than with T2^*^WI (10). This suggests that the SWI is also better at detecting microbleeds in dogs than T2^*^WI. The significance of the visibility of ASV in this weight category should be carefully interpreted because of the small sample size. It is probably due to the fact that this is the group with the most number of “multiple” ASV, reflecting microbleeds. The hypothesis that more intracranial ASV can be detected with SWI in comparison with T2^*^WI only partially withstood statistical testing and had to be partially rejected.

SWI has recently been established in human medicine as the blood-sensitive sequence and is increasingly replacing T2^*^WI (10, 24). The advantages of SWI compared with T2^*^WI include increased susceptibility to hemorrhagic lesions, calcification, and faster acquisition due to the shortened TE. The increased susceptibility may also represent a drawback, as simultaneous amplification of the intrinsic artifacts is enhanced. This phenomenon is proportional to the field strength (18). Although the feasibility of the sequence has been reported in veterinary medicine (1, 3), evaluation of SWI phase images and artifact characterization are currently lacking.

One of the advantages of SWI in comparison with T2^*^WI in humans is increased sensitivity for the detection of small brain parenchymal hemorrhages (11, 12). In dogs, increased conspicuity of hemorrhagic lesions and venous structures has been described (3). While cerebral microbleeds have been defined as ASV under 4 mm in T2^*^WI in dogs (25), this measurement does not represent the true size of the lesion but the size of the blooming artifact produced by the magnetic field distortion. Therefore, a direct comparison of the size of the lesions between the sequences was not performed, and only the detected number of lesions was documented, as the size is variable depending on the magnitude of the signal void and magnet strength.

In addition, SWI has the capability to distinguish hemorrhagic from calcified intracranial lesions based on their magnetic susceptibility (7, 8), a feature that is increasingly implemented in human medicine (7, 9, 26). This application has not yet been tested in dogs and cats. The appearance of paramagnetic and diamagnetic substances displayed on phase images is dependent on the current direction of the magnet. The scanner used in this study is a right-handed magnet; hence, paramagnetic substances, such as hemoglobin decomposition products, display negative phase shifts resulting in reduced signal intensity. Diamagnetic substances, such as calcified tissue, display positive phase shifts, leading to increased signal intensity. Furthermore, the geometry of the lesion and the differences of magnetic susceptibility of the surrounding tissue play a role in the conformation of the signal void (7, 13, 27). In the present study, in more than 95% of the ASV (77/81), phase images were correctly classified as intracranial hemorrhage without a calcified corresponding lesion in the CT scan. A too heterogeneous phase pattern, without definition of a well-defined torus with two lobes, was present in four ASV, preventing a confident classification. In these four lesions, the phase images were not able to make a discrimination between para- or diamagnetic and, hence, between hemorrhage and calcification. The ASV, for which it was not possible to distinguish with certainty between the paramagnetic and diamagnetic characteristics, were heterogeneous and in close proximity to the calvarium. In these cases, parts of the lobe merged with the artifacts originating from the bone. In this study, CT was implemented as the gold standard for detecting intracranial calcified lesions. No calcified lesions were detected in CT. However, correct classification of the vast majority of the hemorrhagic lesions suggests that phase images of the SWI in cats and dogs may be a useful discriminator between blood products and calcification in MRI. Further prospective studies including animals with confirmed calcified intracranial lesions should be performed in order to draw definitive conclusions.

SWI simplifies the detection of hemorrhagic lesions by increasing the conspicuity of susceptibility artifacts. This characteristic also applies to anatomical air–bone interfaces and metallic implants (7, 14, 18). Image distortion originating from the frontal sinuses had the greatest influence in our study regardless of species and weight classification, contradicting our hypothesis that susceptibility artifacts originating from anatomical structures are more prone in animals with low body weight. The size of the frontal sinus and the shape of the frontal bones are widely variable among dog and cat breeds, from absent in brachycephalic breeds to voluminous in dolichocephalic breeds (22, 28, 29). The results show that significantly more severe image distortion is present in dogs with a non-brachycephalic skull conformation at the level of the frontal sinus than in brachycephalic dogs, confirming that skull conformation has an influence on the extent of the artifacts. In a clinical scenario, this might impair lesion detection in the olfactory bulbs and frontal lobes in non-brachycephalic skull conformation. Artifacts from the petrosal portion of the temporal bone are significantly more severe in the whole population of cats in SWI than in the T2^*^WI, but there is no significant difference in the individual subgroups. This suggests that there are generally more artifacts in cats. It remains open if this disadvantage outweighs the benefit of SWI. Image distortion due to the microchip rarely occurs but can seriously limit image evaluation (Supplementary Figure S1).

A limitation due to the retrospective nature of the study is the small number of verified cases, with the classification of the lesion to hemorrhage or calcification in CT. Another limitation is the single-observer approach. A multiple-observer approach could have provided more confidence for the scoring of lesions.

In conclusion, with the advantages of better detection of ASV in SWI compared with T2^*^WI and the opportunity to differentiate between paramagnetic and diamagnetic origin in most cases, SWI is generally recommended for dogs. It must be taken into account that the most limiting factor of SWI is the artifact of the frontal sinus, which is significantly more present in SWI relative to T2^*^WI. Brachycephalic dogs are better to evaluate than non-brachycephalic dogs in the frontal region. Therefore, frontal sinus conformation appears to be a limiting factor in image interpretation.

## Data Availability Statement

The data analyzed in this study is subject to the following licenses/restrictions: patient and owner data are subject to legal regulation. On request, these can be provided anonymized, from the corresponding author. Requests to access these datasets should be directed to Nadja Wolfer, nwolfer@vetclinics.uzh.ch.

## Ethics Statement

Ethical review and approval was not required for the animal study because of the retrospective nature of the study. Written informed consent was obtained from the owners for the participation of their animals in this study.

## Author Contributions

NW, AW-L, and MD contributed to the conception of the study. NW collected the data. NW, MD, AW-L, and KB analyzed the data. HR performed the statistical analysis. NW drafted the manuscript. NW, AW-L, KB, HR, and MD revised and approved the submitted version. All authors contributed to the article and approved the submitted version.

## Conflict of Interest

The authors declare that the research was conducted in the absence of any commercial or financial relationships that could be construed as a potential conflict of interest.

## Publisher's Note

All claims expressed in this article are solely those of the authors and do not necessarily represent those of their affiliated organizations, or those of the publisher, the editors and the reviewers. Any product that may be evaluated in this article, or claim that may be made by its manufacturer, is not guaranteed or endorsed by the publisher.

## Abbreviations

ASV: areas of signal void
DICOM: Digital Imaging and Communications in Medicine
SWI: susceptibility-weighted imaging
T2^*^WI: T2^*^-weighted imaging
T: Tesla
TE: echo time
TR: repetition time.
