# Supplementary material for: Intracranial Lesion Detection and Artifact Characterization: Comparative Study of Susceptibility and T2*-Weighted Imaging in Dogs and Cats
Source: Front Vet Sci. 2021 Dec 13;8:779515. doi: 10.3389/fvets.2021.779515 (PMC8710604; doi:10.3389/fvets.2021.779515)
Supplement: Supplementary file 1 [file Table_1.docx]

Table S1: Comparison of the distribution of presence and absence of areas of signal void (ASV) as well as their number and matches in dogs and cats between the T2*-weighted (T2*WI) and susceptibility-weighted (SWI) sequence.

|  | T2*WI |  | SWI |  | sequences in agreement (pairs) |  |
| --- | --- | --- | --- | --- | --- | --- |
|  | total | dog | total | dog | total | dog |
|  |  | cat |  | cat |  | cat |
| all | 212 | 160 | 212 | 160 |  |  |
|  |  | 52 |  | 52 |  |  |
| absence | 178 | 132 | 175 | 130 | 167 | 124 |
|  |  | 46 |  | 45 |  | 43 |
| presence | 34 | 28 | 37 | 30 | 26 | 22 |
|  |  | 6 |  | 7 |  | 4 |
| Number of ASV |  |  |  |  |  |  |
| 1 | 21 | 15 | 22 | 17 | 11 | 8 |
|  |  | 6 |  | 5 |  | 3 |
| 2 | 4 | 4 | 3 | 1 | 0 |  |
|  |  | 0 |  | 2 |  |  |
| 3 | 2 | 2 | 2 | 2 | 2 | 2 |
|  |  | 0 |  | 0 |  | 0 |
| 4 | 1 | 1 | 0 |  | 0 |  |
|  |  | 0 |  |  |  |  |
| 5 | 1 | 1 | 1 | 1 | 0 |  |
|  |  | 0 |  | 0 |  |  |
| Multiple (>5) | 5 | 5 | 9 | 9 | 5 | 5 |
|  |  | 0 |  | 0 |  | 0 |

Abbreviations. SWI: susceptibility weighted imaging; T2*WI: T2*-weighted imaging; ASV: areas of signal void
