# Supplementary material for: Intracranial Lesion Detection and Artifact Characterization: Comparative Study of Susceptibility and T2*-Weighted Imaging in Dogs and Cats
Source: Front Vet Sci. 2021 Dec 13;8:779515. doi: 10.3389/fvets.2021.779515 (PMC8710604; doi:10.3389/fvets.2021.779515)
Supplement: Supplementary file 2 [file Data_Sheet_1.docx]

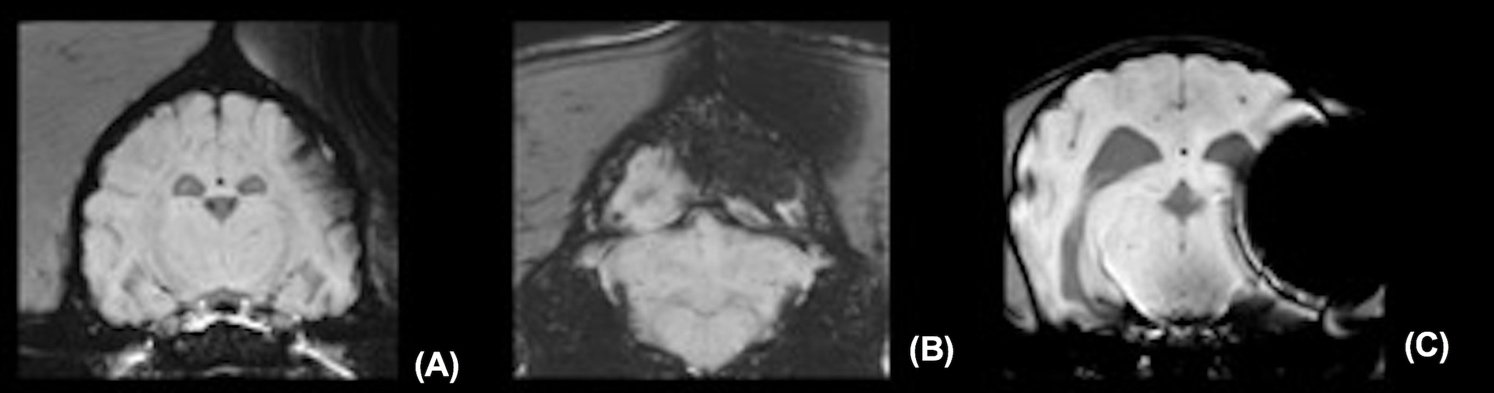


**Supplementary Figure 1.** Different degrees of impairment to image evaluation due to artifacts originating from the microchip in susceptibility-weighted images (SWI) (A mild, B moderate, C severe).
